# Supplementary material for: Cell Type Specific Suppression of Hyper-Recombination by Human RAD18 Is Linked to Proliferating Cell Nuclear Antigen K164 Ubiquitination
Source: Biomolecules. 2025 Jan 20;15(1):150. doi: 10.3390/biom15010150 (PMC11763143; doi:10.3390/biom15010150)
Supplement: Supplementary file 1 [file biomolecules-15-00150-s001.zip › biomolecules-3410098-supplementary.pdf]

SUPPLEMENTARY FIGURE TITLES AND LEGENDS for

Rogers et. al., *Biomolecules*

"Cell type specific suppression of hyper-recombination by human RAD18 is linked to PCNA K164 ubiquitination."

Figure S1. Loss of MLH1 expression does not increase SCEs in hTERT RPE-1 cell lines.

(A) Western blot analysis confirming loss of MLH1 expression in two independent RPE-1 *MLH1*$^{-/-}$ clones and two independent *RAD18*$^{-/-}$/*MLH1*$^{-/-}$ clones, with β-actin as the loading control. The asterisk indicates a nonspecific band recognized by the anti-RAD18 antibody. Quantification is based on densitometry using FIJI normalized to the loading control and WT lane, minus signal from the nonspecific RAD18 band.

(B) Average number of SCEs per chromosome in RPE-1 MLH1-proficient and -deficient cell lines. Black lines indicate mean values and significance was calculated using a Mann-Whitney test. No comparisons were statistically significant.

(C) Original western blot images related to Figure S1A.

Figure S2. Original western blot images related to Figure 1.

(A) Original western blot images related to Figure 1A.

(B) Original western blot images related to Figure 1B.

(C) Original western blot images related to Figure 1C.

(D) Original western blot images related to Figure 1I.

Figure S3. Original western blot images related to Figure 3.

Figure S4. Generation and characterization of HCT116 *PCNA*$^{K164R/+}$ mutant cell lines.

(A) Sanger sequencing trace files from HCT116 *PCNA*$^{K164R/+}$ cell lines. The dotted red box indicates the codon for the K164 residue. The dotted orange or pink boxes indicate the sequences for the diagnostic EcoRI or XcmI restriction enzyme recognition sites, respectively.

(B) (Left) Schematic of *PCNA* in HCT116 *PCNA*$^{K164R/+}$ cells indicating that exon 2 was targeted by CRISPR-Cas9. The locations of the Cas9 cut site and primers used are indicated. Allele-specific frameshift mutations were determined by TOPO-TA cloning followed by Sanger sequencing of PCR amplicons. (Right) Table summarizing results of TOPO-TA cloning and sequencing analyses.

(C) Clonogenic survival of HCT116 wild-type and two *PCNA*$^{K164R/+}$ cell lines following MMS treatment at the indicated dosages (5, 10 or 20 µM). Error is indicated as standard deviation and significance was calculated using two-tailed students *t-test* with * > 0.05; ** > 0.01, *** > 0.001 across at least two biological replicates.

Figure S5. Original western blot and gel images related to Figure 4.

(A) Original gel image related to Figure 4B.

(B) Original western blot images related to Figure 4D.

Figure S6. Original western blot images related to Figure 5.

(A) Original western blot images related to Figure 5A.

(B) Original western blot images related to Figure 5E.

Figure S7. Expression of super-telomerase (ST) effectively lengthens telomeres in *PCNA*$^{K164R/+}$ or reverted *PCNA*$^{+/+}$ cell lines.

(A) Telomere restriction fragment length analyses of *PCNA*$^{K164R/+}$ cell lines (top) or reverted *PCNA*$^{+/+}$ cell lines (bottom) expressing ST. Yellow lines indicate the location of highest average peak intensity. Arrows indicate clones that were used for further studies.

(B) Original TRF images related to Figure S7A.

Figure S1

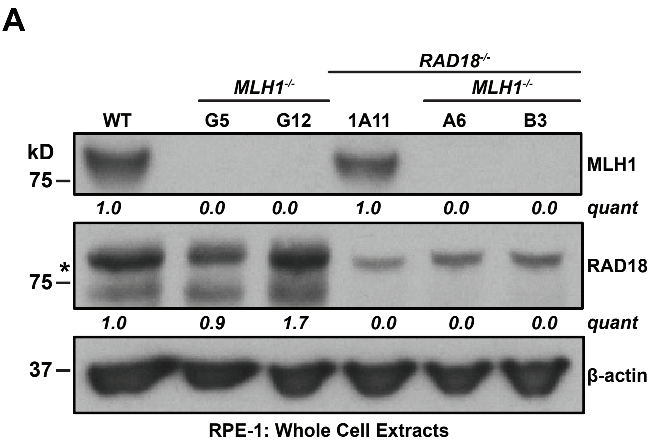
A
RAD18-/-
MLH1-/-
MLH1-/-
WT
G5
G12
1A11
A6
B3
kD
75
MLH1
1.0
0.0
0.0
1.0
0.0
0.0
quant
*
75
RAD18
1.0
0.9
1.7
0.0
0.0
0.0
quant
37
β-actin
RPE-1: Whole Cell Extracts

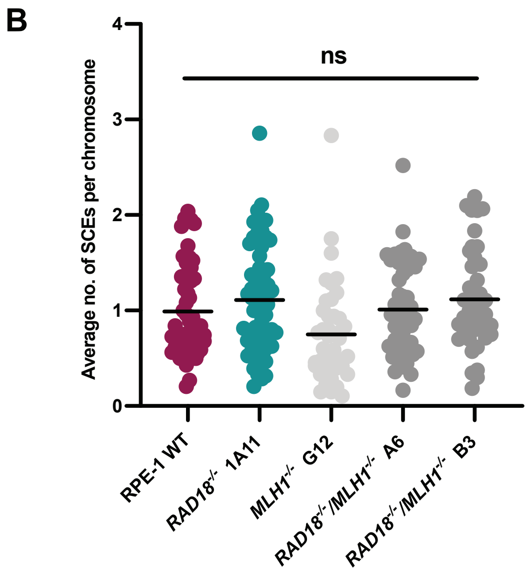
B
ns
Average no. of SCEs per chromosome
0
1
2
3
4
RPE-1 WT
RAD18-/- 1A11
MLH1-/- G12
RAD18-/-/MLH1-/- A6
RAD18-/-/MLH1-/- B3

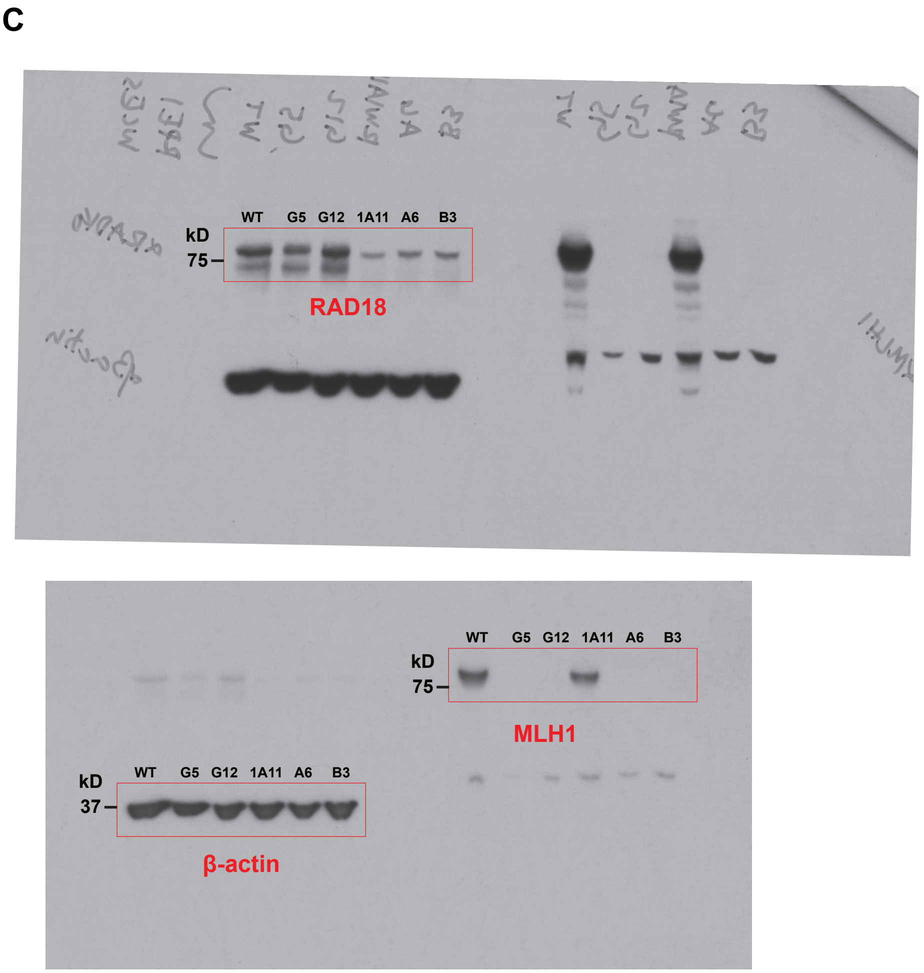
C
WT
G5
G12
1A11
A6
B3
kD
75
RAD18
WT
G5
G12
1A11
A6
B3
kD
75
MLH1
WT
G5
G12
1A11
A6
B3
kD
37
β-actin

Figure S2

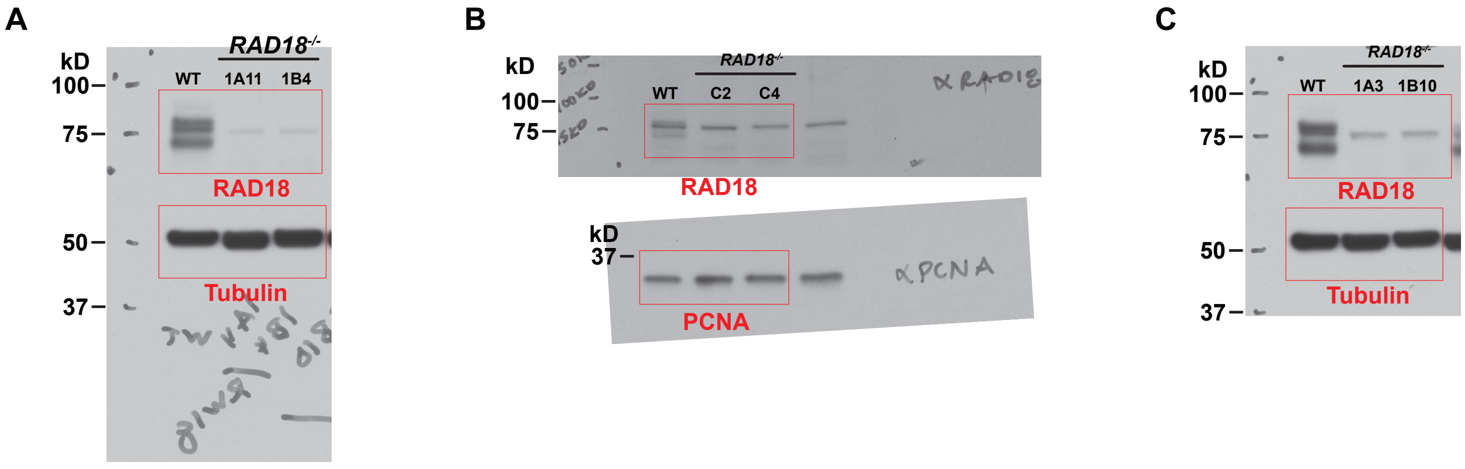
A
kD
100
75
50
37
RAD18-/-
WT
1A11
1B4
RAD18
Tubulin
B
kD
100
75
37
RAD18-/-
WT
C2
C4
RAD18
PCNA
C
kD
100
75
50
37
RAD18-/-
WT
1A3
1B10
RAD18
Tubulin

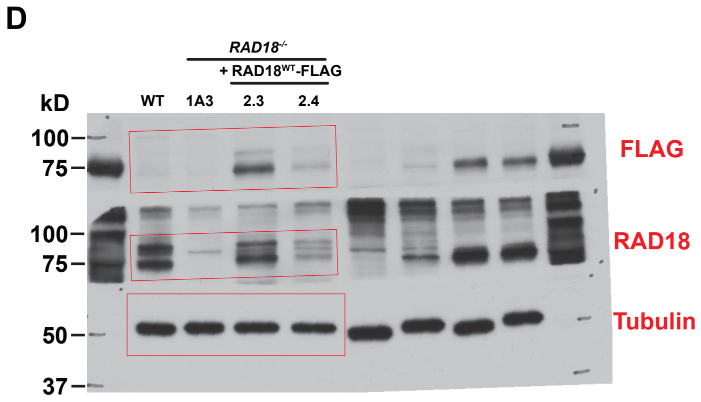
D
RAD18-/-
+ RAD18WT-FLAG
kD
WT
1A3
2.3
2.4
100
75
100
75
50
37
FLAG
RAD18
Tubulin

Figure S3

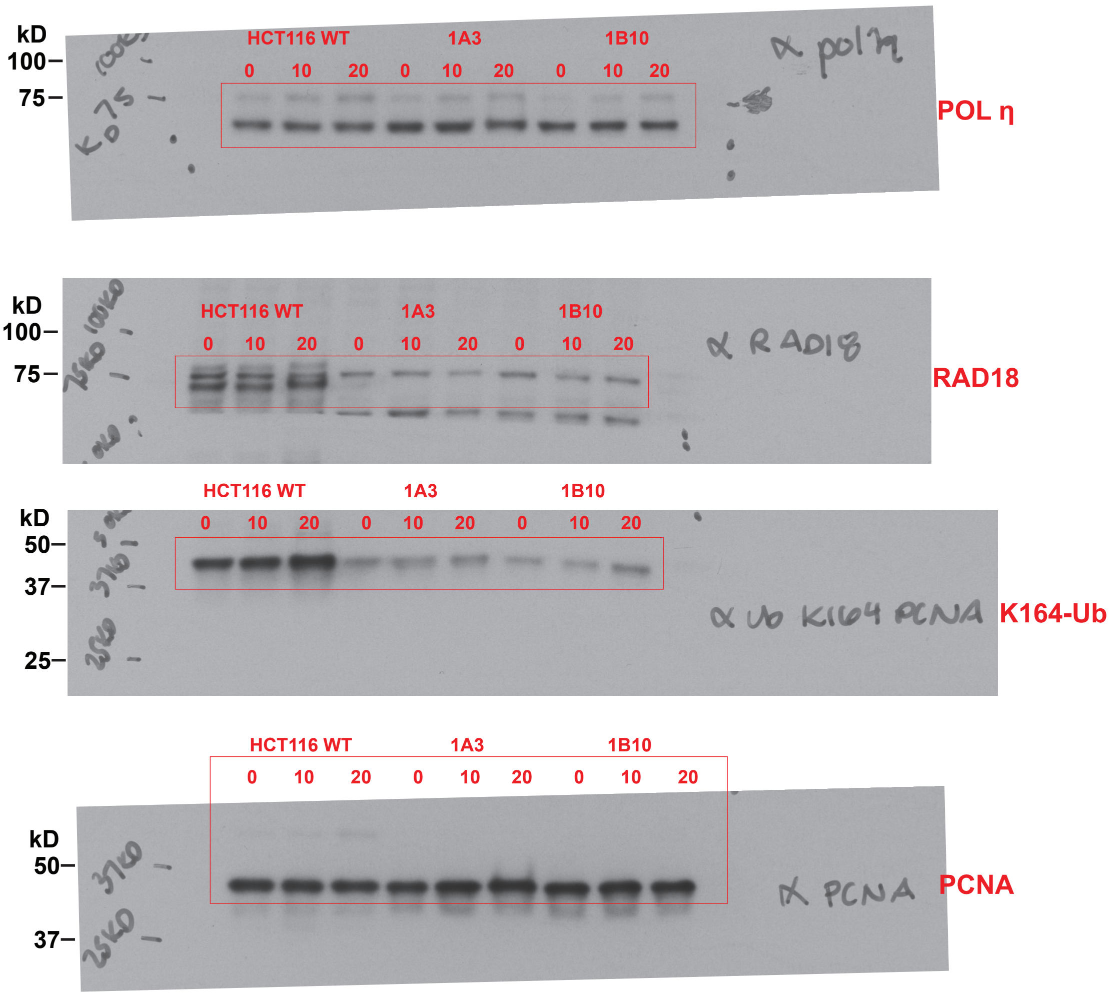
kD
100
75
HCT116 WT
1A3
1B10
0 10 20 0 10 20 0 10 20
POL η
kD
100
75
HCT116 WT
1A3
1B10
0 10 20 0 10 20 0 10 20
RAD18
kD
50
37
25
HCT116 WT
1A3
1B10
0 10 20 0 10 20 0 10 20
K164-Ub
kD
50
37
HCT116 WT
1A3
1B10
0 10 20 0 10 20 0 10 20
PCNA

Figure S4

A

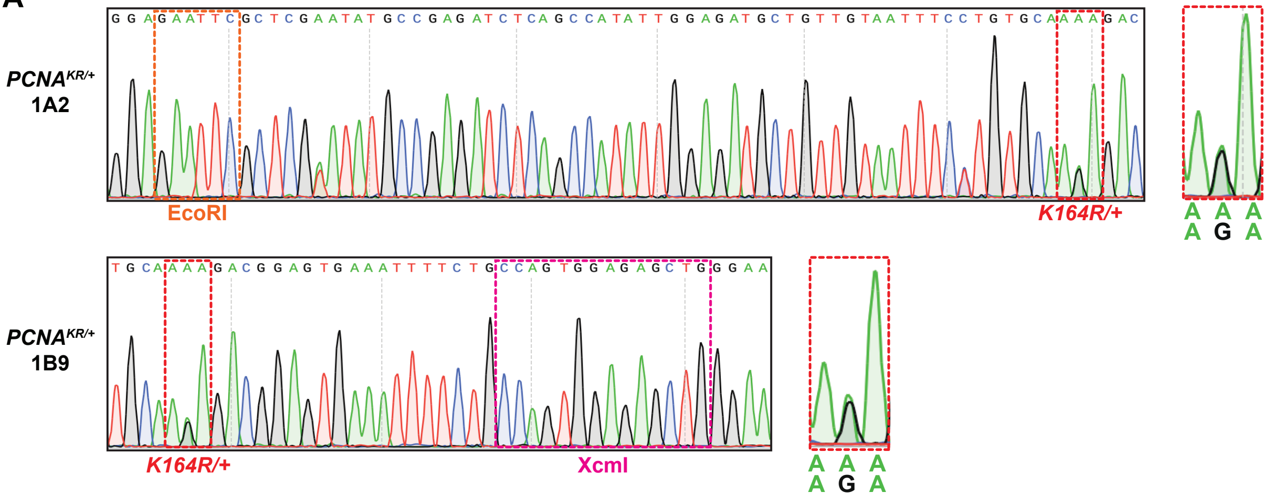

B

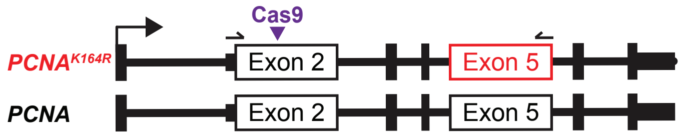

TA Clone #	Exon 2	Exon 5
1	WT	K164R
2	KO	K164R
3	WT	WT
4	KO	K164R
5	WT	WT
6	WT	WT
7	KO	K164R
8	KO	WT
9	WT	K164R

C

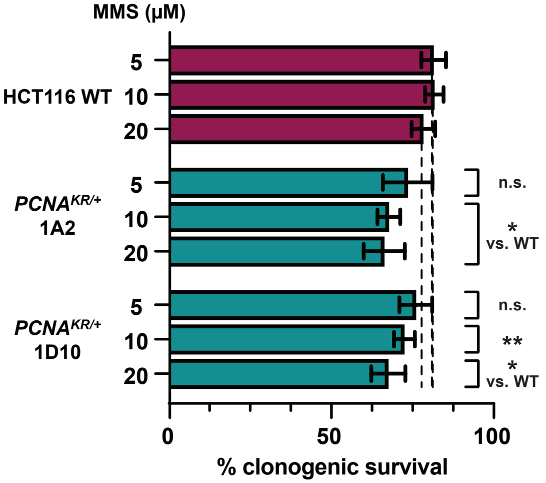

Figure S5

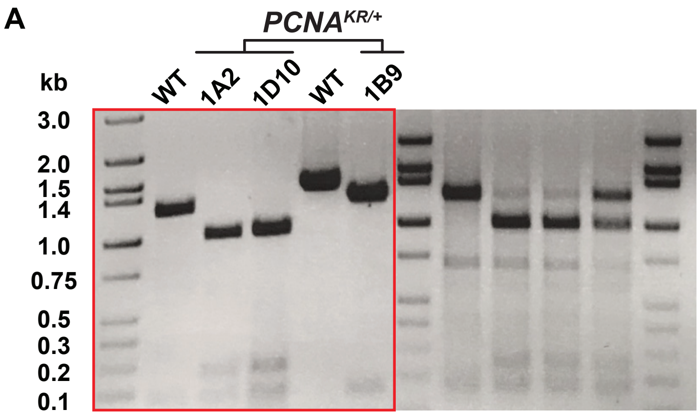
A
PCNA^KR/+
kb
WT
1A2
1D10
WT
1B9
3.0
2.0
1.5
1.4
1.0
0.75
0.5
0.3
0.2
0.1

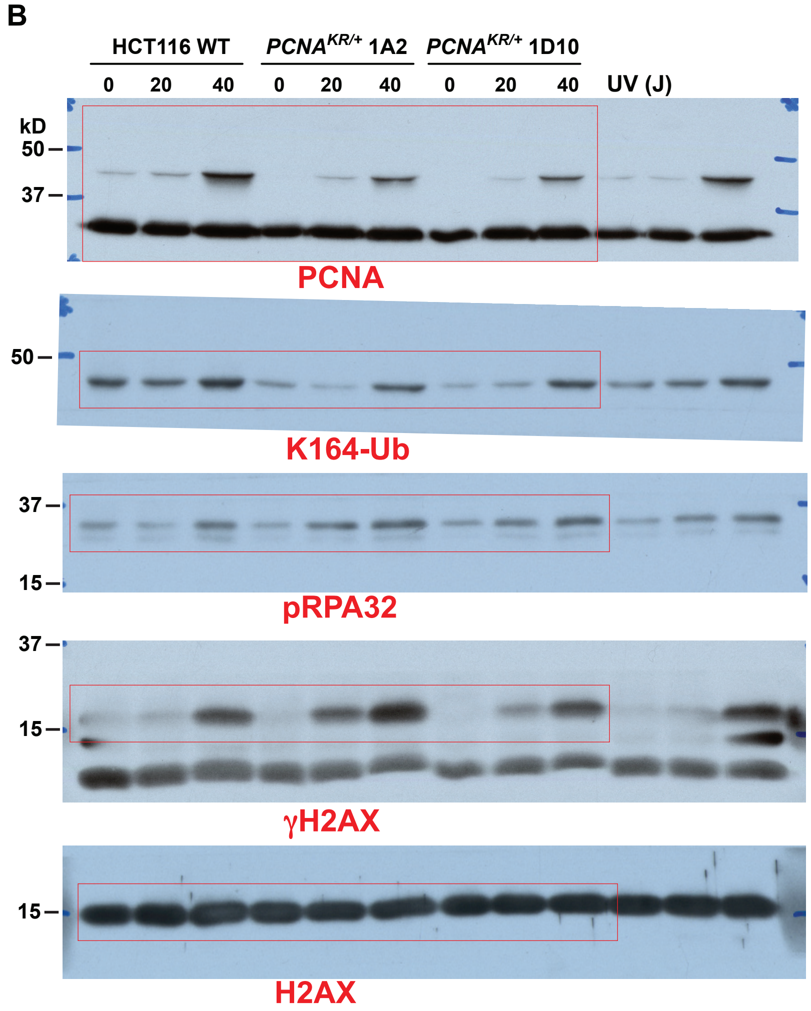
B
HCT116 WT
PCNA^KR/+ 1A2
PCNA^KR/+ 1D10
0 20 40 0 20 40 0 20 40 UV (J)
kD
50
37
PCNA
50
K164-Ub
37
15
pRPA32
37
15
γH2AX
15
H2AX

Figure S6

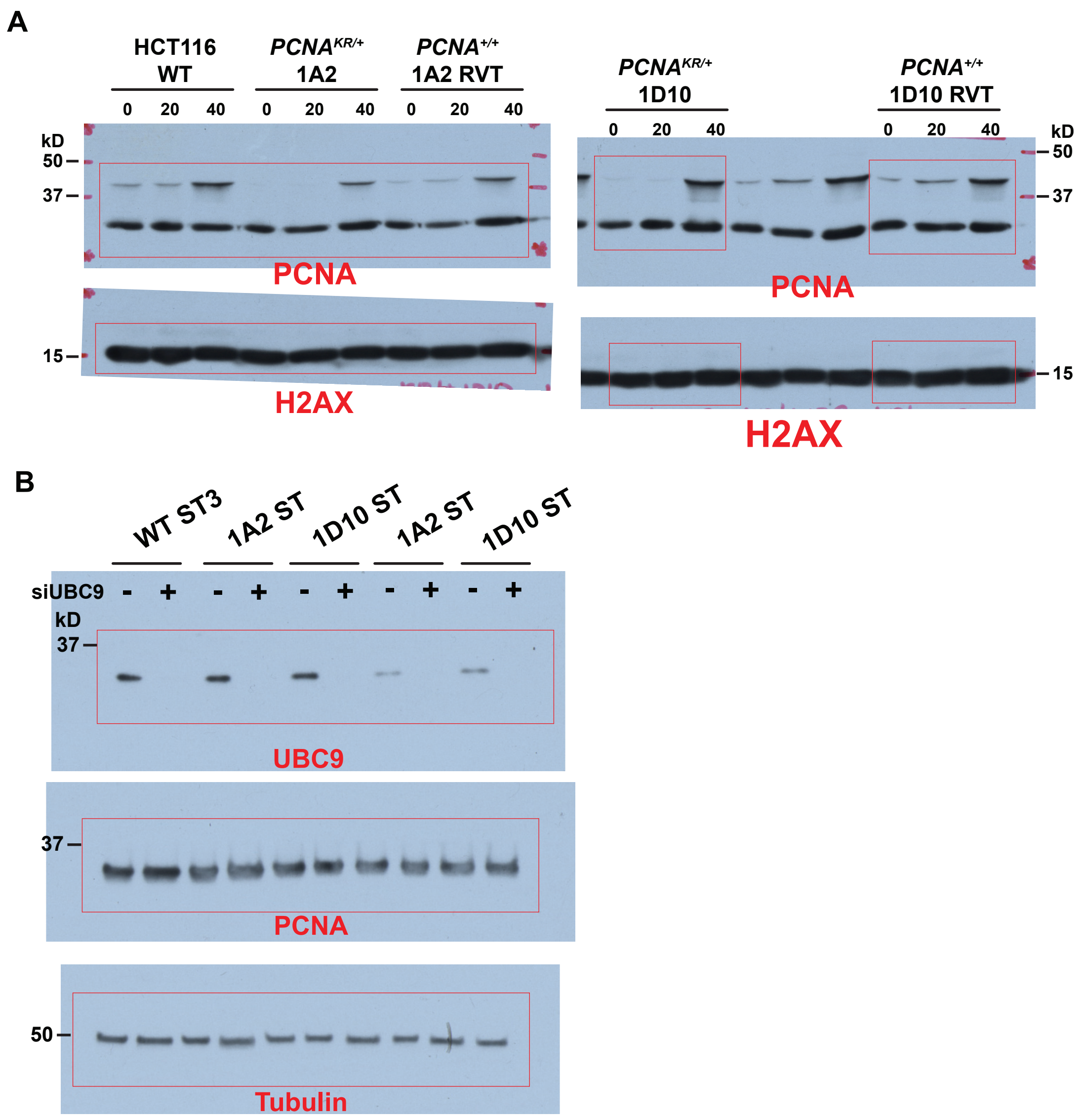
A
HCT116 WT
PCNA^KR/+ 1A2
PCNA^+/+ 1A2 RVT
0 20 40
0 20 40
0 20 40
kD
50
37
PCNA
15
H2AX
PCNA^KR/+ 1D10
PCNA^+/+ 1D10 RVT
0 20 40
0 20 40
kD
50
37
PCNA
15
H2AX
B
WT ST3
1A2 ST
1D10 ST
1A2 ST
1D10 ST
siUBC9 - + - + - + - + - +
kD
37
UBC9
37
PCNA
50
Tubulin

Figure S7

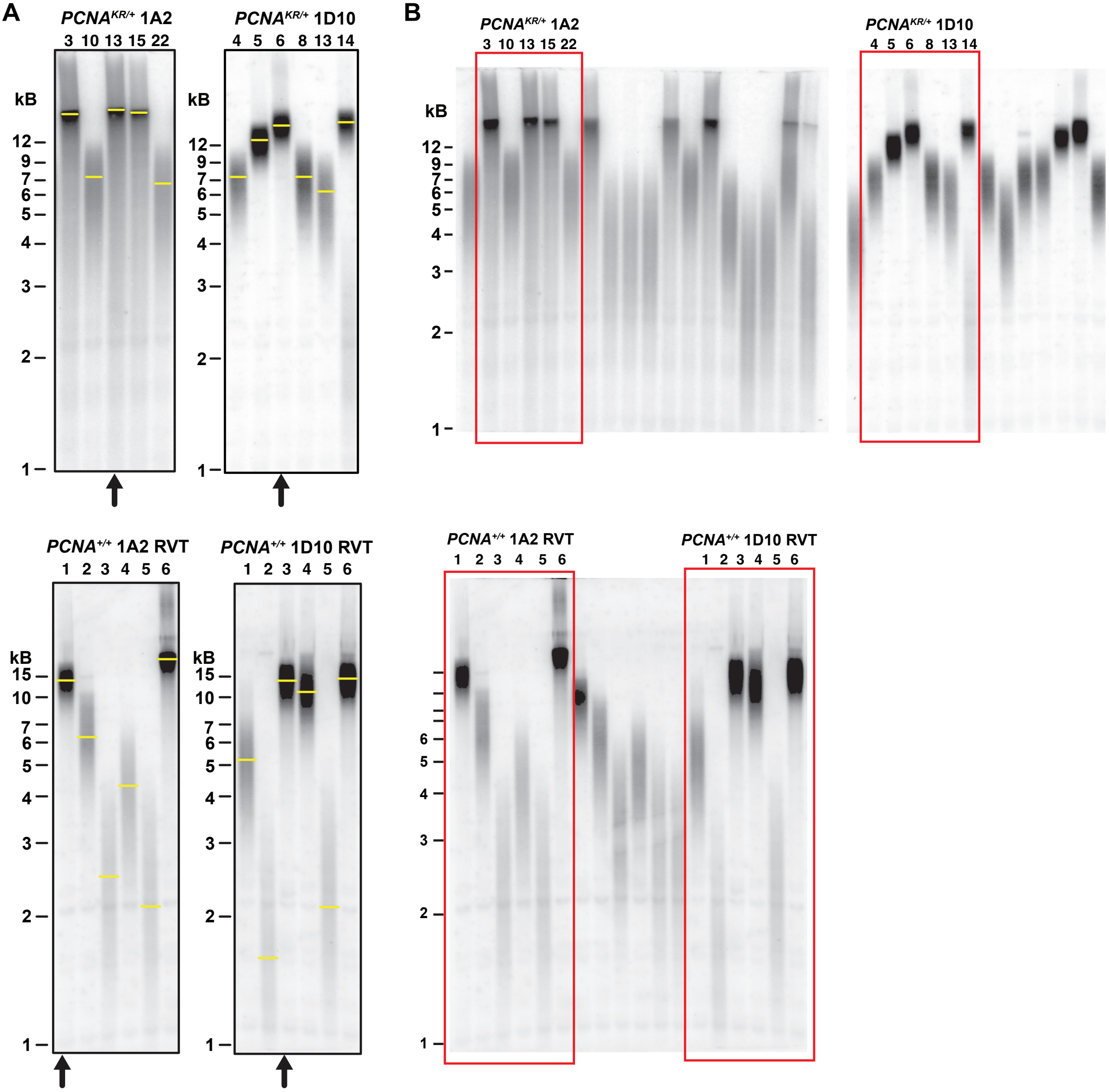
A
B
PCNA^KR/+ 1A2
3 10 13 15 22
PCNA^KR/+ 1D10
4 5 6 8 13 14
kB
12
9
7
6
5
4
3
2
1
PCNA+/+ 1A2 RVT
1 2 3 4 5 6
PCNA+/+ 1D10 RVT
1 2 3 4 5 6
kB
15
10
7
6
5
4
3
2
1
